# Development, Validation, and Subtype Analysis of a Predictive Model for Atrial Fibrillation in Patients With Hypertrophic Cardiomyopathy

**DOI:** 10.31083/RCM45267

**Published:** 2025-11-27

**Authors:** Ailian Shen, Jing Xu, Qiucang Xue, Hongming Yu, Jing Liang, Xiuzheng Yue, Yuying Liu, Dan Mu

**Affiliations:** ^1^Department of Radiology, Nanjing Drum Tower Hospital Clinical College of Jiangsu University, 210008 Nanjing, Jiangsu, China; ^2^Department of Radiology, Nanjing Drum Tower Hospital Clinical College of Nanjing Medical University, 210008 Nanjing, Jiangsu, China; ^3^Department of Radiology, Nanjing Drum Tower Hospital, Affiliated Hospital of Medical School, Nanjing University, 210008 Nanjing, Jiangsu, China; ^4^Philips Healthcare, 100020 Beijing, China; ^5^Laboratory National Key Laboratory for Novel Software Technology, Department of Computer Science and Technology, Nanjing University, 210023 Nanjing, Jiangsu, China; ^6^Department of Radiology, Shanghai Tenth People's Hospital, Tongji University School of Medicine, 200125 Shanghai, China

**Keywords:** hypertrophic cardiomyopathy, atrial fibrillation, cardiac magnetic resonance imaging, risk prediction model, obstructive and non-obstructive subtypes

## Abstract

**Background:**

Atrial fibrillation (AF) is a major complication of hypertrophic cardiomyopathy (HCM) with significant prognostic implications. Current risk prediction models lack the integration of comprehensive cardiac magnetic resonance (CMR) metrics and subtype-specific analyses.

**Methods:**

A retrospective study of 405 HCM patients (86 with AF) was performed from 2019 to 2024. After excluding highly correlated variables (|r| > 0.8), the cohort was split into training and validation sets in a 7:3 ratio. Least Absolute Shrinkage and Selection Operator (LASSO) regression and multivariable logistic regression analyses were used to identify predictors, with model performance assessed via receiver operating characteristic (ROC) curves, calibration plots, and decision curve analysis. Subgroup analyses were conducted for obstructive (HOCM) and non-obstructive (HNCM) subtypes.

**Results:**

Independent predictors of AF in the overall HCM cohort included right atrial diameter anteroposterior (RAD anteroposterior: odds ratio (OR) = 1.819, 95% confidence interval (CI) 1.130–3.007; *p* = 0.016), left ventricular end-systolic volume (LVESV: OR = 0.978, 95% CI 0.963–0.991; *p* = 0.002), septal mitral annular plane systolic excursion (MAPSE septal: OR = 0.850, 95% CI 0.736–0.976; *p* = 0.023), tricuspid annular plane systolic excursion (TAPSE: OR = 0.919, 95% CI 0.852–0.987; *p* = 0.022), and maximum left atrial volume (MaxLAV: OR = 1.016, 95% CI 1.004–1.029; *p* = 0.010). The model achieved an area under the curve (AUC) value of 0.850 in the training set and an AUC of 0.861 in the validation set. The HOCM subtype predictors included septal MAPSE and left atrial ejection fraction (LAEF); meanwhile, the HNCM predictors included septal MAPSE, maximal left atrial volume (MaxLAV), and right atrial ejection fraction (RAEF).

**Conclusions:**

A validated multiparametric CMR model can accurately predict AF risk in HCM patients, with subtype-specific predictors enabling personalized monitoring and early intervention.

## 1. Introduction 

Atrial fibrillation (AF) is a common and clinically important complication in hypertrophic cardiomyopathy (HCM), occurring in about 20%–25% of patients and associated with a poorer prognosis due to increased risks of thromboembolism and heart failure [1, 2, 3]. Timely diagnosis and management are critical, as early anticoagulation and rhythm control can significantly lower stroke risk and prevent heart failure progression. While AF is usually identified by standard electrocardiography (ECG) or Holter monitoring after symptoms appear, these methods often detect the arrhythmia only when it is already sustained or symptomatic, leading to delays in intervention. Existing models for predicting AF risk, such as the HCM-AF risk calculator, depend mainly on baseline clinical factors and standard echocardiographic measures—particularly left atrial size and left ventricular wall thickness [4, 5, 6]. Although useful, these models cannot fully capture subclinical atrial remodeling, early systolic or diastolic dysfunction, or myocardial fibrosis, all key factors in AF development. Their dependence on conventional echocardiography also limits evaluation of complex structural and tissue-level changes that contribute to AF in HCM.

Cardiac magnetic resonance (CMR) has become the reference standard for comprehensive assessment of cardiac structure, function, and tissue characteristics in HCM. It allows accurate quantification of left ventricular mass, ejection fraction, and fibrosis through late gadolinium enhancement (LGE) [7], while feature tracking (FT) CMR provides more detailed myocardial strain analysis than echocardiography [8]. With its ability to perform multiparametric evaluation of left and right heart remodeling, CMR offers deeper insights into AF mechanisms and improves prediction [9, 10, 11, 12]. Recent evidence highlights the value of specific CMR-derived measures, including maximal left atrial volume (MaxLAV) and mitral annular plane systolic excursion (MAPSE), as strong predictors of AF. These parameters can identify early systolic impairment before a reduction in left ventricular ejection fraction (LVEF) occurs [13, 14, 15, 16]. Given the frequent presence of asymmetric hypertrophy in HCM, MAPSE is particularly relevant for assessing septal function [14, 15].

Notably, the role of right heart dynamics in HCM-related AF remains understudied [17, 18, 19, 20, 21]. Reduced tricuspid annular plane systolic excursion (TAPSE) and abnormal TAPSE-to-pulmonary artery systolic pressure (PASP) ratios, indicative of impaired right ventricular-pulmonary artery coupling, have been linked to AF susceptibility [19]. HCM patients with AF also exhibit unique patterns of right atrial dilation and TAPSE reduction [15], suggesting a critical yet overlooked contribution of right heart remodeling to AF pathogenesis.

Obstructive (HOCM) and nonobstructive (HNCM) HCM subtypes display distinct hemodynamic profiles, fibrosis distributions, and atrial remodeling patterns [20, 21, 22]. HOCM patients experience elevated left atrial afterload due to outflow tract obstruction, accelerating atrial fibrosis [23], whereas HNCM is associated with right atrial structural changes secondary to myocardial hypertrophy [24]. Critically, existing AF risk models lack subtype-specific analyses, failing to account for the heterogeneous mechanisms underlying AF in HOCM versus HNCM. This limitation hinders precise risk stratification and personalized management.

This study aimed to: (1) identify key CMR-derived parameters associated with AF in patients with HCM; and (2) develop and validate a novel risk score based on these parameters specifically for predicting AF risk in this patient population. Additionally, we sought to explore the differential contributions of HOCM and HNCM phenotypes to atrial remodeling and AF risk.

## 2. Materials and Methods 

### 2.1 Research Subjects 

This retrospective study included 405 patients with HCM who underwent CMR and were hospitalized at Nanjing Gulou Hospital between January 2019 and December 2024. The main admission diagnoses of these patients covered categories: chest pain, dyspnea, palpitations, syncope, or routine follow-up for HCM. The study protocol was approved by the Institutional Ethics Committee (2024-551-01) with a waiver for informed consent.

Inclusion Criteria (Based on the 2024 American Heart Association guidelines [21]): (a) Septal thickness ≥15 mm (or ≥13 mm in the presence of a family history of HCM, confirmed by imaging modalities including echocardiography, CMR, or computed tomography (CT)); (b) Age ≥18 years.

Exclusion Criteria: (a) Age <18 years; (b) Incomplete clinical or imaging data; (c) Poor image quality, defined as significant artifacts (e.g., motion artifacts, susceptibility artifacts), missing frames, or insufficient resolution that impeded accurate assessment of cardiac structures; (d) Severe comorbidities, including active infection, malignancy, renal or hepatic dysfunction, acute myocardial infarction; (e) Infiltrative or systemic cardiomyopathies and other conditions associated with secondary hypertrophy, including refractory hypertension, aortic stenosis, and other infiltrative cardiomyopathies.

### 2.2 Definition of AF in HCM Patients

AF was diagnosed according to the 2023 American College of Cardiology/American Heart Association/American College of Chest Physicians/Heart Rhythm Society (ACC/AHA/ACCP/HRS) AF diagnostic guidelines [25]. The diagnostic criteria were: (a) ECG findings: A single-lead ECG recording for ≥30 seconds or a 12-lead ECG for ≥10 seconds demonstrating abnormal atrial waveforms, characterized by the absence of P waves, presence of irregular fibrillatory waves (f-waves) with variable size, shape, and timing, accompanied by irregular RR intervals; (b) AF status: Only patients diagnosed with AF during the current hospital admission were included, without further subclassification into paroxysmal or persistent forms. Patients with a pre-existing AF diagnosis prior to the current admission were excluded to focus on new-onset AF during hospitalization.

### 2.3 CMR Data Acquisition and Measurement

**Fig. 1.**
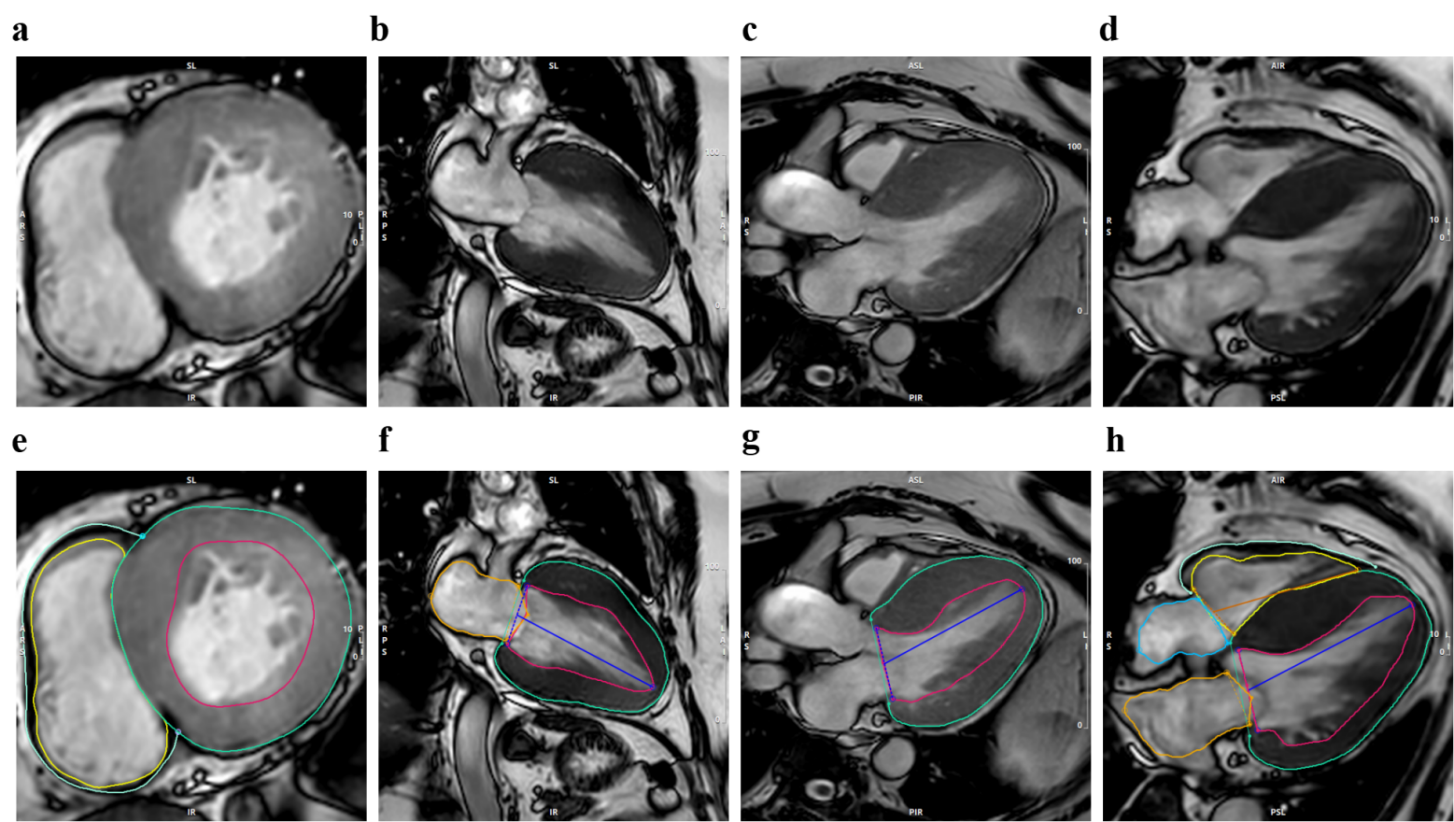
**Typical cardiac magnetic resonance imaging (CMRI) presentation in patients with hypertrophic cardiomyopathy (HCM)**. This figure shows representative cardiac MRI images and contoured images obtained from patients with HCM, utilizing cinematic MRI technology to precisely depict the structural and functional characteristics of the heart in various anatomical planes. Where (a–d) correspond to the short-axis (SAX), two-chamber (2CH), three-chamber (3CH), and four-chamber (4CH) views of the original cinematic MRI, respectively; (e–h) corresponding to the SAX, 2CH, 3CH, and 4CH views of the cinematic MRI contoured in the original image, respectively.

CMR scans were performed during hospitalization using a 3.0-T MRI system (Philips Healthcare, Best, Netherlands) in accordance with standardized imaging protocols recommended by the Society for Cardiovascular Magnetic Resonance (SCMR) [26]. All scans were completed during sinus rhythm to ensure accurate measurement of cardiac volume and functional parameters. Scans were acquired at end-expiration using vector ECG-gated techniques with the patient in the supine position. A balanced turbo field echo (B-TFE) movie sequence was used to capture two-, triple-, and four-lumen long-axis views of the left ventricle (LV), as well as successive short-axis slices. For T1 mapping imaging, a breath-hold modified MOLLI (Modified Look-Locker Inversion Recovery) sequence was employed to acquire three short-axis slices at the basal, midventricular, and apical levels. Enhanced T1 measurements were performed 10–15 minutes after intravenous administration of 0.2 mmol/kg gadolinium-based contrast agent (Magnevist, Bayer Healthcare, Berlin, Germany). Images were then translated to the hospital’s Picture Archiving and Communication System (PACS). All CMR analyses were independently conducted by two experienced radiologists using PACS and CVI42 software (version 6.1, Circle Cardiovascular Imaging, Alberta, Canada) (Fig. 1).

**Cardiac structure**: right atrial diameter (RAD) anteroposterior, LA diameter (LAD) anteroposterior, left ventricular wall thickness (LVWT), and interventricular septum thickness (IVST) were measured using the short cine image.

**Functional parameters**: MAPSE (assessing left ventricular longitudinal systolic function, including inferior, anterior, lateral, and septal walls) and TAPSE (assessing right ventricular systolic function)—were measured using two- and four-chamber cine images.

**Other parameter**: LV and RV overall strain—global circumferential strain (GCS), global longitudinal strain (GLS), and global radial strain (GRS)—measured by FT-CMR. Pre-contrast T1 values, post-contrast T1 values, and extracellular volume (ECV) were obtained using the tissue T1 mapping function (see **Supplementary Table 1** for the specific parameter list).

### 2.4 Model Building Process

In order to avoid the limitations of traditional models that rely solely on 2D ultrasound, this study integrated clinical baseline data with multi-dimensional CMR parameters to develop a predictive model through the following steps:

#### 2.4.1 Data Preprocessing

Variables with more than 10% missing values were eliminated (e.g., T1 mapping-related parameters were excluded due to high rates of missing data) (**Supplementary Table 2**). Multicollinear variables were removed based on Pearson correlation analysis, retaining only one variable from each group of highly correlated variables (|r| > 0.8 was considered a strong correlation) (**Supplementary Table 2**).

#### 2.4.2 Dataset Splitting

Patients were randomly divided into a training set (model development) and a validation set in a 7:3 ratio to ensure that the baseline characteristics of the two groups—such as age, gender, and incidence of AF—were balanced.

#### 2.4.3 Feature Selection

In the training set, Least Absolute Shrinkage and Selection Operator (LASSO) regression was applied. Specifically, 10-fold cross-validation was performed, and the optimal regularization parameter λ was selected by minimizing the mean squared error using the 1-SE rule variables with non-zero coefficients that were significantly associated with AF were filtered out.

#### 2.4.4 Model Building

Variables selected through LASSO regression were incorporated into a multivariable logistic regression model. Predictors with a *p*-value less than 0.05 were considered significant and retained, leading to the construction of a prediction model that included both clinical and radiological indicators. Subsequently, the risk scores from this model were visualized using a nomogram.

### 2.5 Model Evaluation and Subgroup Analysis

#### 2.5.1 Performance Evaluation

Discrimination was assessed using the receiver operating characteristic curve (AUC) and the concordance index (C-index). The agreement between predicted probabilities and actual outcomes was evaluated through calibration curves generated with 1000 bootstrap resamples. Additionally, decision curve analysis (DCA) was performed to assess the net benefit across various risk thresholds, thereby enabling the evaluation of the model’s clinical utility.

#### 2.5.2 Subgroup Analysis 

Stratification was based on LVOT gradient (≥30 mmHg vs. <30 mmHg) [27].

Each subgroup underwent the same modeling pipeline, ensuring methodological consistency. This approach enables identification of subtype-specific predictors and quantifies how obstruction modifies predictive performance.

### 2.6 Statistical Analysis

All statistical analyses were conducted using R software (version 4.3.2, R Foundation for Statistical Computing, Vienna, Austria). Continuous variables were summarized as mean ± standard deviation (Mean ± SD) for normally distributed data, or as median (M) and interquartile range (Q1–Q3) for non-normally distributed data. Categorical variables were presented as counts and percentages. Normality was assessed using the Shapiro-Wilk test. Between-group comparisons were performed using independent sample *t*-tests or Mann-Whitney U tests for continuous variables, and chi-square or Fisher’s exact tests for categorical variables, as appropriate. All statistical tests were two-sided, with *p* < 0.05 considered statistically significant.

## 3. Results 

### 3.1 Patient Characteristics

**Fig. 2.**
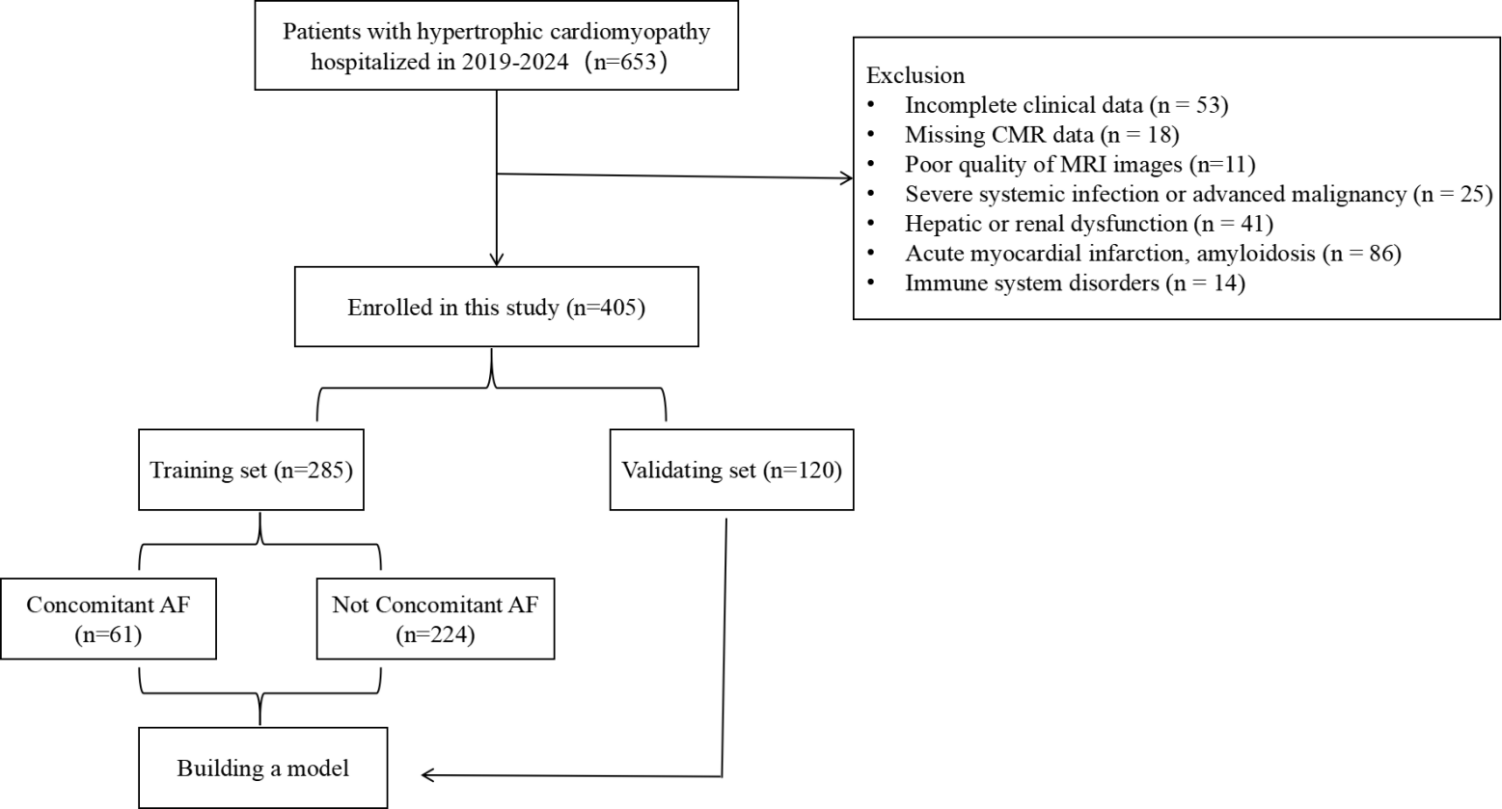
**Flow chart for patient selection**. AF, Atrial Fibrillation.

This study enrolled 405 patients with HCM, and the participant screening process was illustrated in (Fig. 2). The baseline characteristics of the AF and non-AF groups are as follows: (1) Significant differences were observed between the two groups regarding gender, age, eosinophil count, aspartate aminotransferase (AST), lactate dehydrogenase (LDH), direct bilirubin, cholinesterase, urea, creatinine, estimated glomerular filtration rate (eGFR), free triiodothyronine (FT3), and free thyroxine (FT4) (all *p* < 0.05) (Table 1); For CMR parameters, significant intergroup differences between AF and non-AF groups were observed in all measured indices except LVWT, left ventricular end-systolic volume (LVESV), and right ventricular end-systolic volume (RVESV) (all *p* < 0.05; Table 2). The incidence of AF showed no significant difference between the training and validation sets (21.4% vs. 20.8%, *p* > 0.05), with comparable baseline demographics and CMR metrics across groups (*p* values 0.052–1.000) (**Supplementary Table 4**). Notable exceptions were MAPSE Septal (*p* = 0.035), MAPSE Anterior (*p* = 0.016), and MAPSE Inferior (*p* = 0.034) (**Supplementary Table 4**). Such intergroup variations in segmental MAPSE potentially compromised the model’s generalizability in the validation set; however, LASSO regression was applied to exclude MAPSE features with significant intergroup differences and minimal AF relevance, thereby mitigating the model’s dependency on training-set-specific distributions and enhancing its clinical applicability.

**Table 1.** **Baseline features of the dateset**.

Variables		Total (n = 405)	No_AF (n = 319)	AF (n = 86)	*p*-value
CHD, n (%)					
	Yes	73 (18%)	57 (18%)	16 (19%)	1.000
Obstruction, n (%)					
	Yes	154 (38%)	125 (39%)	29 (34%)	0.423
Gender, n (%)					
	Male	263 (65%)	217 (68%)	46 (53%)	0.017
Age		57 (48, 66)	56 (46, 66)	63 (55, 69)	<0.001
Heart rate (times/minute)		69 (62, 76)	69 (62, 76)	69 (61, 77)	0.814
Hypertension, n (%)					
	Yes	218 (54%)	172 (54%)	46 (53%)	1.000
Diabetes, n (%)					
	Yes	48 (12%)	34 (11%)	14 (16%)	0.214
Alcohol, n (%)					
	Yes	75 (19%)	60 (19%)	15 (17%)	0.894
Smoking, n (%)					
	Yes	129 (32%)	109 (34%)	20 (23%)	0.072
BMI		25.77 ± 3.62	25.75 ± 3.62	25.85 ± 3.65	0.811
White blood cell (10^9^/L)		6.1 (5.1, 7.4)	6.1 (5.2, 7.4)	5.8 (5.03, 7.35)	0.457
NLR		2.00 (1.58, 2.80)	2.05 (1.57, 2.82)	2.00 (1.59, 2.71)	0.902
Monocyte (10^9^/L)		0.4 (0.3, 0.5)	0.4 (0.3, 0.5)	0.4 (0.3, 0.5)	0.278
Eosinophil (10^9^/L)		0.13 (0.08, 0.20)	0.13 (0.08, 0.21)	0.11 (0.07, 0.18)	0.043
Basophil (10^9^/L)		0.03 (0.02, 0.04)	0.03 (0.02, 0.04)	0.03 (0.02, 0.04)	0.895
Red blood cell (10^12^/L)		4.59 (4.23, 4.95)	4.6 (4.23, 4.96)	4.54 (4.22, 4.93)	0.555
Hemoglobin (g/L)		140 (128, 152)	141 (128, 153)	139 (128, 151)	0.460
Platelet (10^9^/L)		188 (150, 228)	191 (153, 233)	185 (147, 220)	0.278
Alanine aminotransferase (U/L)		20.1 (14.6, 29.1)	19.9 (14.5, 28.8)	20.55 (15.05, 29.77)	0.619
Aspartate aminotransferase (U/L)		21.1 (17.8, 25.8)	20.6 (17.4, 25.1)	23.6 (19, 29.0)	0.002
Alkaline phosphatase (U/L)		69.3 (56.2, 80.4)	69.5 (56.3, 81.1)	66.6 (55.8, 77.7)	0.510
Glutamyl transpeptidase (U/L)		27.6 (18.9, 44.3)	27.2 (18.8, 42.7)	30.3 (19.3, 45.7)	0.299
Lactate dehydrogenase (U/L)		200 (176, 234)	197 (175, 226)	220.6 (185.3, 262.5)	0.001
Direct bilirubin (umol/L)		2.4 (1.8, 3.3)	2.4 (1.75, 3.22)	2.8 (2.0, 4.4)	0.003
Cholinesterase (KU/L)		7.9 (6.7, 9.1)	8.0 (6.9, 9.3)	7.7 (6.1, 8.3)	0.012
Albumin (g/L)		40.8 (38.7, 42.4)	40.9 (38.8, 42.4)	40.2 (38.3, 42.1)	0.091
Globulin (g/L)		24.9 (21.9, 27.4)	25.1 (21.9, 27.6)	23.9 (21.9, 26.2)	0.099
Leucine aminopeptidase (U/L)		43.25 (29.6, 51.5)	43.25 (29.65, 51.50)	43.18 (29.45, 51.03)	0.901
Adenosine deaminase (U/L)		9.7 (7.8, 12.1)	9.8 (7.8, 12.0)	9.6 (7.6, 12.3)	0.890
Urea (mmol/L)		6 (4.9, 7.1)	5.8 (4.8, 7.0)	6.5 (5.2, 7.5)	0.016
Creatinine (umol/L)		70 (59, 80)	69 (58, 78)	74.5 (64.3, 83.8)	0.004
Triglyceride (mmol/L)		1.26 (0.91, 1.79)	1.25 (0.91, 1.81)	1.28 (0.89, 1.71)	0.691
HDL-C (mmol/L)		1.06 (0.90, 1.25)	1.06 (0.90, 1.25)	1.11 (0.90, 1.27)	0.365
LDL-C (mmol/L)		2.51 ± 0.80	2.53 ± 0.79	2.41 ± 0.82	0.207
C-reactive protein (mg/L)		3.5 (2.3, 5.5)	3.3 (2.3, 5.5)	3.85 (2.4, 5.5)	0.352
eGFR (mL/min/1.73 m^2^)		101.2 (83.9, 118.4)	104.4 (88.4, 122.6)	90.0 (71.5 101.4)	<0.001
TSH (mLU/L)		1.99 (1.34, 2.74)	1.97 (1.34, 2.71)	2.02 (1.35, 3.05)	0.515
Free triiodothyronine (pmoL/L)		4.66 (4.20, 5.11)	4.73 (4.27, 5.18)	4.53 (4.05, 4.86)	0.008
Free thyroxine (pmoL/L)		16.5 (14.8, 18.1)	16.5 (14.6, 18.1)	17.0 (15.6, 18.4)	0.033

CHD, Coronary Atherosclerotic Heart Disease; NLR, neutrophil-to-lymphocyte ratio; HDL-C, High-Density Lipoprotein Cholesterol; LDL-C, Low-Density Lipoprotein Cholesterol; eGFR, estimated Glomerular Filtration Rate; TSH, Thyroid Stimulating Hormone.

**Table 2.** **CMR parameter characteristics of the dateset**.

Variables	Total (n = 405)	No_AF (n = 319)	AF (n = 86)	*p*-value
RAD anteroposterior (cm)	3.3 (3.0, 3.9)	3.3 (2.9, 3.7)	3.7 (3.2, 4.6)	<0.001
LAD anteroposterior (cm)	3.3 (2.8, 3.9)	3.2 (2.6, 3.6)	4.2 (3.4, 4.6)	<0.001
LVWT (cm)	1.0 (0.8, 1.2)	1.0 (0.8, 1.2)	0.9 (0.7, 1.17)	0.103
IVST (cm)	1.9 (1.6, 2.3)	1.9 (1.6, 2.2)	2.1 (1.7, 2.3)	0.017
LVEDV (mL)	130.26 (112.18, 155.93)	134.98 (114.1, 161.79)	122.34 (107.02, 142.07)	<0.001
LVESV (mL)	53.94 (41.05, 67.95)	53.94 (40.84, 67.28)	54.30 (42.56, 68.64)	0.682
LVEF	58.69 (51.34, 64.51)	59.39 (53.53, 64.96)	53.97 (46.36, 61.24)	<0.001
LVCI (L/min/m^2^)	2.74 (2.26, 3.27)	2.77 (2.30, 3.33)	2.61 (2.18, 2.92)	0.004
RVEDV (mL)	112.12 (94.43, 136.40)	117.06 (95.30, 137.80)	103.18 (88.75, 127.76)	0.008
RVESV (mL)	57.07 (44.23, 73.80)	56.82 (44.26, 73.47)	58.94 (44.32, 74.22)	0.786
RVEF	50.11 (37.66, 58.31)	51.32 (39.34, 58.90)	46.52 (32.41, 53.89)	0.004
RVCO (L/min)	3.69 (2.67, 4.78)	3.81 (2.80, 5.01)	3.26 (2.41, 4.26)	0.008
RVCI (L/min/m^2^)	2.05 (1.47, 2.62)	2.07 (1.51, 2.66)	1.92 (1.30, 2.39)	0.031
MAPSE inferior (mm)	9.88 ± 3.77	10.44 ± 3.78	7.81 ± 2.93	<0.001
MAPSE anterior (mm)	9.45 (7.00, 11.54)	9.81 (7.58, 11.90)	7.33 (5.44, 9.84)	<0.001
MAPSE lateral (mm)	12.75 (10.02, 15.03)	13.20 (10.87, 15.39)	10.61 (8.65, 13.15)	<0.001
MAPSE septal (mm)	8.93 ± 3.37	9.50 ± 3.31	6.83 ± 2.71	<0.001
TAPSE (mm)	17.77 (13.72, 21.62)	18.60 (15.30, 22.36)	13.45 (10.06, 17.85)	<0.001
LVGLS	–11.9 (–14.6, –9.0)	–12.47 (–14.80, –9.50)	–9.75 (–12.38, –7.12)	<0.001
LVGRS	27.8 ± 9.41	28.54 ± 9.24	25.05 ± 9.57	0.003
RVGCS	–13.44 (–16.20, –10.47)	–13.55 (–16.39, –10.81)	–12.47 (–15.03, –9.38)	0.029
RVGLS	–23.51 (–26.62, –18.58)	–23.87 (–27.05, –19.22)	–20.70 (–24.97, –16.63)	0.001
MaxLAV (mL)	83.3 (62.41, 109.47)	76.53 (59.68, 99.03)	115.7 (83.14, 151.93)	<0.001
MaxLAA (cm^2^)	24.38 (20.49, 29.25)	23.15 (19.89, 27.45)	30.63 (25.18, 35.69)	<0.001
LAEF	49.74 (39.82, 57.91)	52.49 (45.25, 59.37)	35.07 (21.93, 45.12)	<0.001
MinRAA (cm^2^)	11.50 (9.05, 14.46)	11.19 (8.66, 13.54)	14.36 (10.87, 19.35)	<0.001
MaxRAV (mL)	57.52 (44.98, 73.92)	55.05 (43.66, 72.17)	66.95 (53.72, 84.22)	<0.001
MaxRAA (cm^2^)	19.02 (16.56, 22.60)	18.68 (15.99, 21.97)	20.97 (18.27, 25.31)	<0.001
RAEF	48.56 (39.68, 57.27)	50.28 (41.08, 58.43)	42.39 (30.50, 49.53)	<0.001

LV, Left Ventricle; RV, Right Ventricle; LA, Left Atrium; RA, Right Atrium; RAD, Right Atrial Diameter; LAD, Left Atrial Diameter; LVWT, Left Ventricular Wall Thickness; IVST, Interventricular Septum Thickness; EDV, End-Diastolic Volume; ESV, End-Systolic Volume; EF, Ejection Fractions; CO, Cardiac Output; CI, Cardiac Index; MAPSE, Mitral Annular Plane Systolic Excursion; TAPSE, Tricuspid Annular Plane Systolic Excursion; GCS, Global Circumferential Strain; GLS, Global Longitudinal Strain; GRS, Global Radial Strain; LAV, LA Volume; LAA, LA Area; RAV, RA Volume; RAA, RA Area; LVESV, Left Ventricular End-Systolic Volume.

### 3.2 Overall Cohort: Key Predictors and Performance Validation of the AF Prediction Model

#### 3.2.1 Independent Predictors

**Fig. 3.**
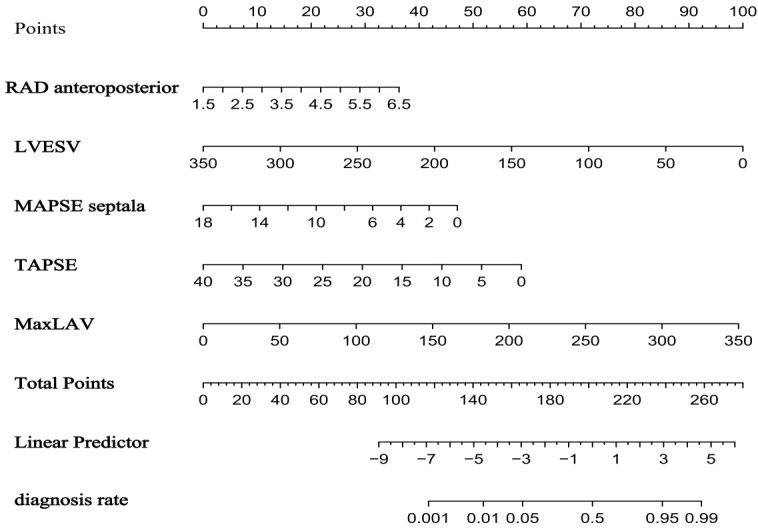
**Nomogram for predicting atrial fibrillation risk in hypertrophic cardiomyopathy based on the training set**.

To further address the issue of multicollinearity, the research employed LASSO regression for feature selection in the training dataset. Through 10-fold cross-validation (K = 10), the optimal regularization parameter (λ = 0.0525301) was determined by minimizing the mean squared error (MSE) and applying the 1-SE rule, ultimately retaining 8 features with non-zero coefficients (**Supplementary Fig. 1a,b**). A multivariate logistic regression model was then constructed based on these features, and the results identified five variables as independent predictors of AF in patients with HCM: RAD Anteroposterior (OR = 1.819, 95% CI: 1.130–3.007, *p* = 0.016), LVESV (OR = 0.978, 95% CI: 0.963–0.991, *p* = 0.002), MAPSE Septal (OR = 0.850, 95% CI: 0.736–0.976, *p* = 0.023), TAPSE (OR = 0.919, 95% CI: 0.852–0.987, *p* = 0.022), and MaxLAV (OR = 1.016, 95% CI: 1.004–1.029, *p* = 0.010) (Table 3). A nomogram was constructed based on these predictors to facilitate clinical risk assessment (Fig. 3).

**Table 3.** **Multivariate logistic regression analysis of predicting AF risk in HCM on training set**.

Variable	OR	95% CI	*p*-value
Age	1.023	0.993–1.056	0.143
eGFR	0.991	0.976–1.006	0.239
RAD Anteroposterior	1.819	1.130–3.007	0.016
LVESV	0.978	0.963–0.991	0.002
MAPSE Septal	0.850	0.736–0.976	0.023
TAPSE	0.919	0.852–0.987	0.022
MaxLAV	1.016	1.004–1.029	0.010
LAEF	0.965	0.929–1.002	0.067

#### 3.2.2 Evaluation of Model Performance and Clinical Utility

**Fig. 4.**
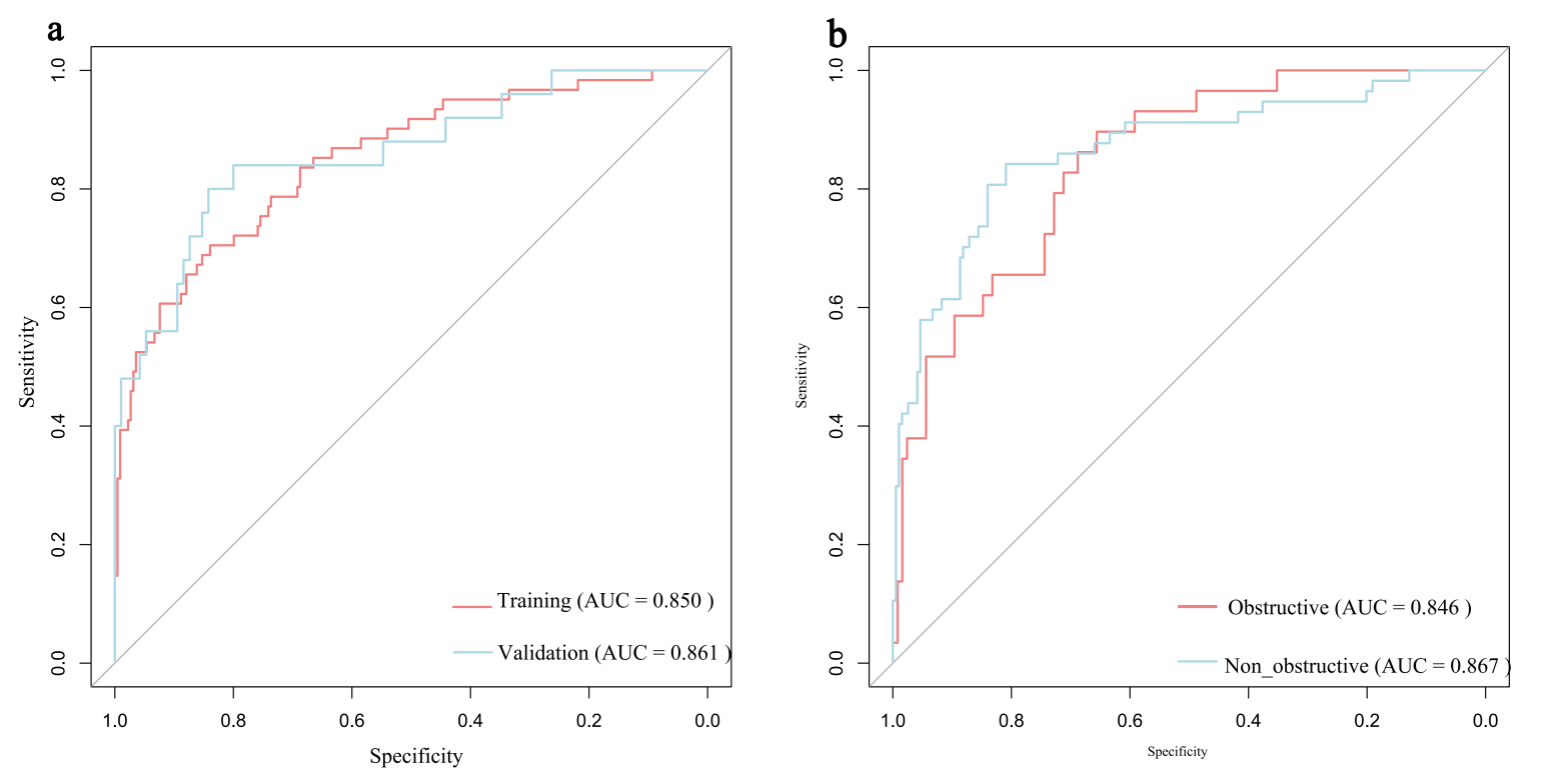
**ROC curves for predicting AF risk in HCM**. (a) ROC curve in the training set and the validation set. (b) ROC curve in the HOCM cohort and the HNCM cohort. HOCM, Hypertrophic Obstructive Cardiomyopathy; HNCM, Hypertrophic Non-Obstructive Cardiomyopathy.

**Fig. 5.**
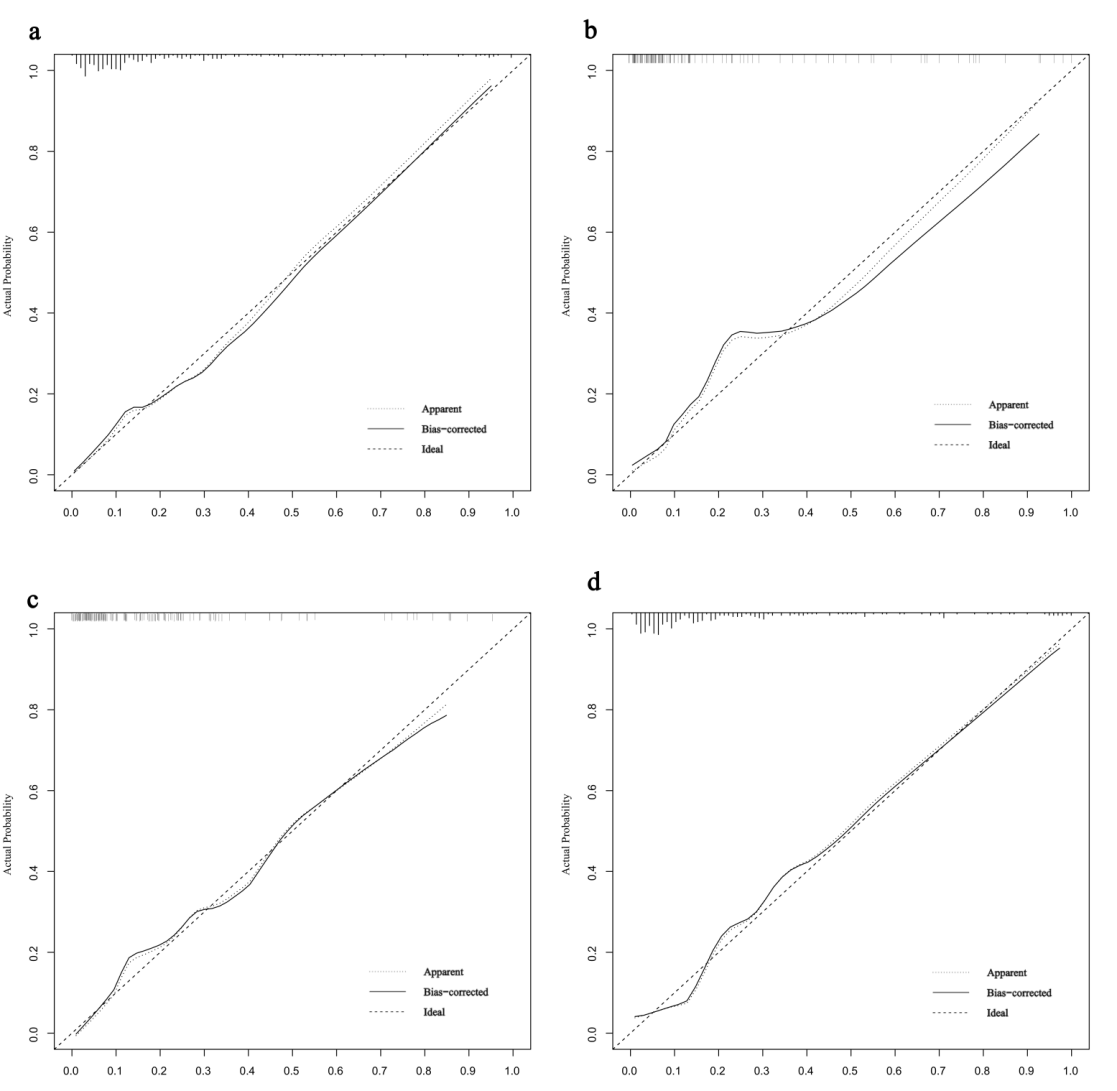
**Calibration curves for predicting AF risk in HCM**. (a) Calibration curve in the training set. (b) Calibration curve in the validation set. (c) Calibration curve in the HOCM cohort. (d) Calibration curve in the HNCM cohort.

**Fig. 6.**
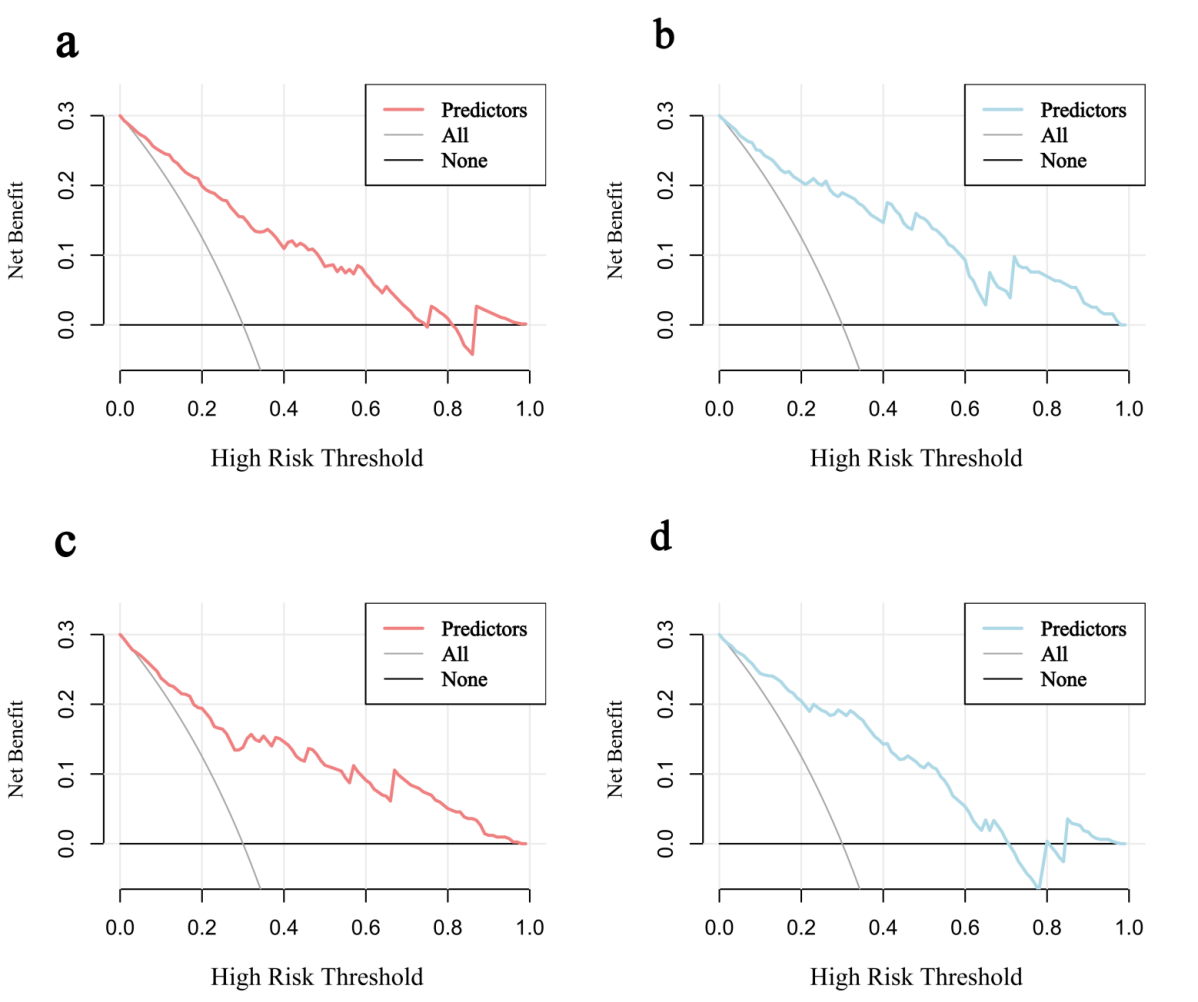
**Decision curves for predicting AF risk in HCM**. (a) Decision curve in the training set. (b) Decision curve in the validation set. (c) Decision curve in the HOCM cohort. (d) Decision curve in the HNCM cohort.

The model demonstrated robust discriminative ability, with a AUC/C-index of 0.850 in the training set and an AUC of 0.861 (95% CI: 0.771–0.952) in the validation set (Fig. 4a). Calibration was assessed using the Hosmer-Lemeshow test, yielding non-significant *p*-values for both the training (*p* = 0.944) and validation (*p* = 0.836) sets, indicating excellent agreement between predicted and observed probabilities. Calibration curves closely approximated the ideal diagonal line (Fig. 5a,b). DCA revealed that the model provided net clinical benefit across a wide range of threshold probabilities (Fig. 6a,b). Specifically, the model outperformed both the “treat-all” and “treat-none” strategies within the threshold range of 10%–65%, suggesting optimal clinical utility for patients with moderate pretest probability. These findings underscore the model’s potential to inform personalized clinical decision-making.

### 3.3 Subgroup Analysis of HOCM and HNCM

#### 3.3.1 Obstructive Versus Non-Obstructive Subgroup Features

This study categorized patients into HOCM (n = 154, 38%) and HNCM (n = 251, 62%) based on left ventricular outflow tract obstruction. The HNCM group did not exhibit a significantly higher incidence of AF than the HOCM group (22.7% vs. 18.8%, *p* = 0.423 (**Supplementary Table 5**)). Additionally, given the potential disparities in anatomical structure and hemodynamics between obstructive and non-obstructive HCM, significant differences were observed between the two groups in baseline and CMR parameter characteristics, such as MaxLAV (*p* = 0.006) (**Supplementary Table 5**).

#### 3.3.2 Independent Predictors

LASSO regression was employed for variable screening in HOCM and HNCM. The optimal λ values of 0.07528455 for HOCM and 0.07574634 for HNCM were determined by minimizing the MSE and applying the 1-SE rule (**Supplementary Fig. 1**).

HOCM: MAPSE septal (OR = 0.844, 95% CI: 0.710–0.991, *p* = 0.044) and LAEF (OR = 0.900, 95% CI: 0.856–0.940, *p* < 0.001) (Table 4).

**Table 4.** **Multivariate logistic regression analysis of predicting AF risk in HCM on HOCM cohort and HNCM cohort**.

Cohort	Variable	OR	95% CI	*p*-value
HOCM	MAPSE Septal	0.844	0.710–0.991	0.044
	LAEF	0.900	0.856–0.940	<0.001
HNCM	RAD Anteroposterior	1.315	0.783–2.229	0.303
	LAD Anteroposterior	1.164	0.562–2.412	0.682
	MAPSE Septal	0.838	0.718–0.971	0.022
	MaxLAV	1.018	1.002–1.036	0.042
	LAEF	0.968	0.927–1.009	0.122
	RAEF	0.960	0.926–0.992	0.020

HNCM: MAPSE septal (OR = 0.838, 95% CI: 0.718–0.976, *p* = 0.022), MaxLAV (OR = 1.018, 95% CI: 1.002–1.036, *p* = 0.042), and right atrial ejection fraction (RAEF, OR = 0.960, 95% CI: 0.926–0.992, *p* = 0.020) (Table 4).

#### 3.3.3 Subgroup Model Performance Evaluation

**Fig. 7.**
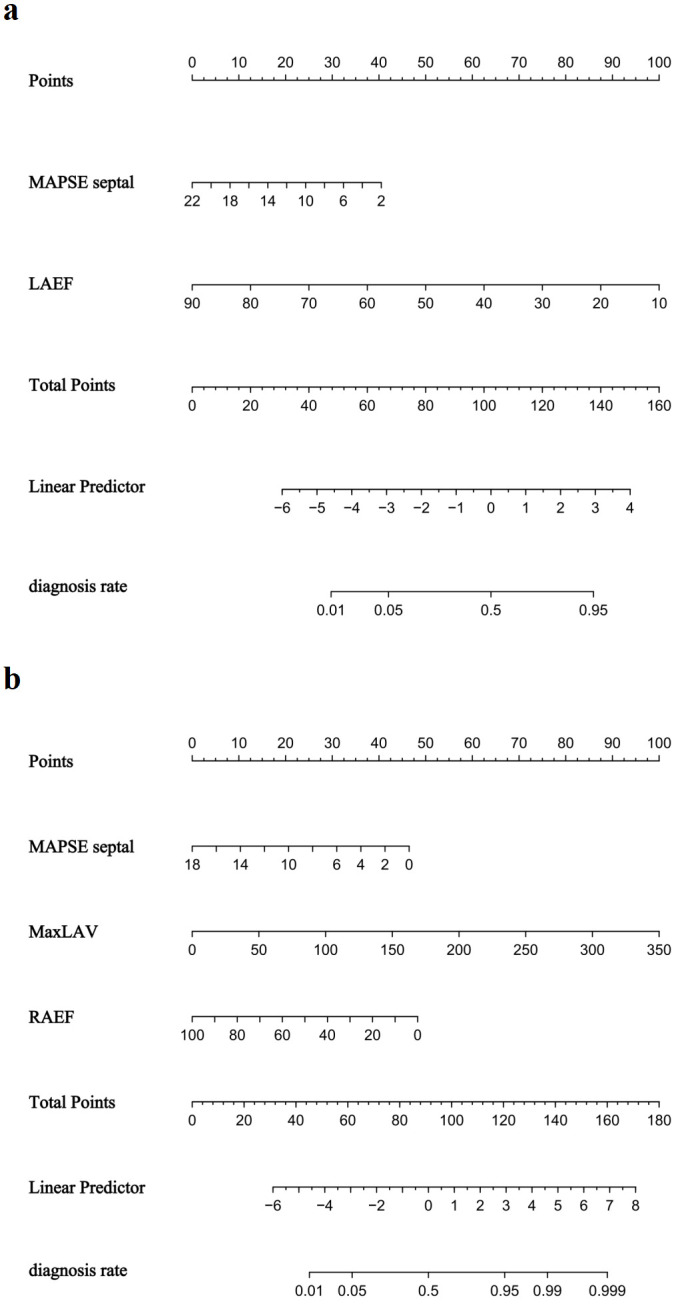
**Nomogram for predicting atrial fibrillation risk in hypertrophic cardiomyopathy**. (a) HOCM cohort. (b) HNCM cohort.

Nomograms were constructed for HOCM (Fig. 7a) and HNCM (Fig. 7b), yielding AUCs of 0.846 and 0.867, respectively (ROC curves shown in Fig. 4b). Calibration curves verified close alignment between predicted and observed probabilities (Fig. 5c,d). DCA revealed that subtype-specific models conferred greater net clinical benefit across their respective risk thresholds compared to “treat-all” or “treat-none” strategies (Fig. 6c,d).

## 4. Discussion 

This study integrated CMR parameters and clinical data to develop an AF prediction model for HCM patients, overcoming the limitations of traditional echocardiography-based models. The model showed excellent performance in the validation set (AUC = 0.861), confirming that CMR-derived myocardial strain and atrial remodeling indices can detect early systolic dysfunction missed by LVEF. Moreover, CMR provides superior tissue characterization, including fibrosis assessment via LGE, which is not feasible with echocardiography. This allows for earlier identification of patients at high risk for AF, even before overt clinical symptoms or ECG changes occur. By differentiating between HOCM and HNCM subtypes, the study revealed distinct AF pathological mechanisms driven by hemodynamic heterogeneity. These findings establish CMR as central to precise AF monitoring in HCM and provide imaging evidence for targeted rhythm control strategies. Thus, incorporating CMR into routine HCM workup can facilitate proactive management and personalized intervention, ultimately improving clinical outcomes.

### 4.1 Development of a Risk Prediction Model for HCM With AF

In recent years, the field of AF risk prediction in cardiomyopathy has witnessed substantial advancements. Multiple studies [28, 29, 30, 31] highlight the pivotal role of CMR in evaluating left heart structure/function, predicting AF onset, and guiding clinical management. Marstrand *et al*. [32] have confirmed that reduced left ventricular ejection fraction (LVEF) is strongly associated with increased AF risk in HCM, a finding mechanistically linked to hemodynamic imbalance and atrial remodeling secondary to systolic dysfunction. Additionally, Schmidt *et al*. [33] reveal a correlation between LVESV and AF via Mendelian randomization analysis. Building on these findings, our study introduced LVESV as a sensitive marker of early systolic reserve in HCM, overcoming LVEF’s limitation of relying on dynamic ejection fraction changes to enable earlier detection of systolic dysfunction. Notably, left atrial enlargement serves as a key prognostic factor for AF recurrence after ablation, with MaxLAV acting as an independent predictor (OR = 1.016, 95% CI: 1.004–1.029, *p* = 0.010) [13]—consistent with den Uijl *et al*. [34], who identify left atrial volume (LA) as the strongest independent predictor of AF recurrence (OR = 1.019, *p* = 0.018). By integrating LVESV with atrial remodeling indices, our study addresses a critical gap in traditional models (e.g., HCM-AF calculator) [4, 5] by incorporating CMR-derived ventricular-atrial coupling markers, thus providing a more comprehensive AF risk assessment framework.

The characteristic asymmetric left ventricular hypertrophy in HCM significantly impairs interventricular septum function. As a core parameter for evaluating left ventricular longitudinal systolic function [14], MAPSE septal exhibits distinct abnormalities in HCM. Doesch *et al*. [15] have demonstrated that septal MAPSE is significantly lower in HCM patients compared to healthy controls (1.57 ± 0.24 vs. 1.31 ± 0.25; *p* = 0.012). Our multiparametric CMR analysis further revealed that septal MAPSE served as an independent predictor of AF (OR = 0.850, 95% CI: 0.736–0.976, *p* = 0.023) in the overall HCM cohort, with differential predictive values in HOCM and HNCM subtypes—findings that underscore the utility of dynamic MAPSE changes for AF risk stratification.

This study innovatively integrated RAD anteroposterior and TAPSE into the predictive model, highlighting the right heart’s independent role in AF pathogenesis. Prabhu *et al*. [35] show that electrical/structural remodeling in the RA correlates with LA remodeling in AF patients, supporting interatrial crosstalk. Our data showed that RAD anteroposterior was a significant AF predictor (OR = 1.82, 95% CI 1.13–3.01, *p* = 0.016), while TAPSE independently predicted AF risk (OR = 0.919, 95% CI 0.852–0.987, *p* = 0.022). Mechanistically, reduced TAPSE reflects impaired right ventricular-pulmonary artery (RV-PA) coupling, which exacerbates RA pressure load and triggers AF [36]—a mechanism supported by a retrospective study showing that lower TAPSE/PASP ratios increase AF risk [19]. Doesch *et al*. [15] further validate the association between decreased TAPSE and right atrial dilation in AF patients. This integration of right heart parameters confirms the RV-PA coupling→RA load→AF pathway, offering a novel paradigm for comprehensive risk stratification in HCM.

### 4.2 Subgroup Analysis Reveals Differences in AF Mechanisms Between HOCM and HNCM Patients

HOCM patients exhibit significantly restricted left ventricular diastolic function due to elevated left ventricular outflow tract gradient (LVOTG ≥30 mmHg). This hemodynamic abnormality drives left atrial dilation and fibrosis by increasing left atrial afterload [18, 34]. This study demonstrated that LAEF, a core indicator of left atrial active emptying capacity, is independently associated with AF occurrence (OR = 0.900, *p* < 0.001), possibly involving compensatory decline in left atrial contractile function with electromechanical delay [6, 37]. Notably, hemodynamic disorders caused by interventricular septal hypertrophy and altered mitral valve spatial relationships in HOCM significantly reduce MAPSE Septal [23, 38]. In contrast, HNCM patients lack significant left ventricular outflow tract obstruction but still exhibit impaired diastolic function and mild hemodynamic abnormalities due to myocardial hypertrophy and ventricular remodeling [24, 39]. These alterations can lead to persistent elevation in right atrial pressure, promoting right atrial dilation and fibrosis, thereby reducing RAEF [24, 39, 40]. This “mild hemodynamic disturbance—right atrial remodeling” pathway is consistent with the mechanism proposed by Croon *et al*. [40], who reported that RAEF combined with RVEF influences AF risk in genetic variant carriers of HCM. Conversely, HOCM patients demonstrate more pronounced left atrial remodeling due to elevated afterload, while right atrial dysfunction may be partially compensated by adaptive mechanisms [21, 38]. These findings collectively highlight the need for obstruction status-based differential clinical assessment: HNCM requires focused monitoring of right atrial structure and function, as mild hemodynamic disturbances gradually accumulate into right atrial remodeling and AF; HOCM necessitates prioritizing optimization of left atrial-right ventricular hemodynamic coupling to interrupt atrial electrophysiological abnormalities from pressure overload. This mechanism-based stratified assessment strategy provides precise imaging and functional targets for personalized AF prevention in HCM patients.

### 4.3 Clinical Implications and Applicability of the Risk Score 

The HCM-AF Risk Score we developed offers clinicians a straightforward tool to stratify AF risk in HCM patients during standard CMR assessments. By integrating parameters already routinely captured in most CMR protocols—such as LVESV and RAEF—this score requires no extra scanning time or additional costs to implement, enhancing its real-world usability. We see particular value in applying this score across three key clinical scenarios: First, it could help flag high-risk patients who might benefit from more rigorous rhythm monitoring (e.g., prolonged Holter monitoring or implantable loop recorders) to catch AF earlier. Second, it may support shared decision-making when anticoagulation prophylaxis is being considered for patients with ambiguous clinical indications. Third, it could refine pre-procedural risk assessment ahead of surgeries like myectomy, where undiagnosed AF might significantly influence perioperative care plans. Of course, broader adoption hinges on future validation. Prospective testing in multi-center cohorts and research exploring how this score impacts actual patient outcomes will be critical to integrating it into routine clinical practice.

### 4.4 Limitations

First, the study’s single-center retrospective design may limit the external validity of the conclusions. Further validation through multicenter prospective studies is required to confirm generalizability. Second, the absence of long-term follow-up data means that the model’s clinical prognostic value remains unestablished. Future studies incorporating longitudinal follow-up are necessary to evaluate its predictive utility for clinical outcomes. Third, the exclusion of patients with pre-existing AF and the focus on in-hospital new-onset AF may limit the generalizability of our findings to the broader HCM population with prevalent or historical AF. Fourth, this study did not include data on mitral regurgitation or tricuspid regurgitation, which are known to be associated with atrial fibrillation and are particularly relevant in obstructive HCM due to their impact on left atrial remodeling and hemodynamic load. Future studies incorporating valvular assessment would enhance the comprehensiveness of AF risk prediction models.

## 5. Conclusion 

This study developed and validated a novel CMR-based risk score that effectively predicts the risk of incident atrial fibrillation in patients with HCM. Independent predictors identified include RAD Anteroposterior, LVESV, MAPSE Septal, TAPSE, and MaxLAV. The model exhibited robust predictive performance in both the training and validation cohorts, with AUC values of 0.850 and 0.861, respectively. Subgroup analysis further revealed mechanistic heterogeneity in AF development between HOCM and HNCM. These findings establish an imaging-based framework for individualized AF risk stratification in HCM patients, offering potential clinical utility for early intervention and personalized management.

## Data Availability

The datasets used in this study are available from the corresponding author upon reasonable request.

## Abbreviations

AF, Atrial fibrillation; HCM, Hypertrophic cardiomyopathy; CMR, Cardiac magnetic resonance; HOCM, Obstructive hypertrophic cardiomyopathy; HNCM, Non-obstructive hypertrophic cardiomyopathy; RAD, Right atrial diameter; LVESV, Left ventricular end-systolic volume; MAPSE, Mitral annular plane systolic excursion; TAPSE, Tricuspid annular plane systolic excursion; MaxLAV, Maximum left atrial volume; LAEF, Left atrial ejection fraction; RAEF, Right atrial ejection fraction.

## Supplementary Material

Supplementary material associated with this article can be found, in the online version, at https://doi.org/10.31083/RCM45267.
