# Supplementary material for: Development, Validation, and Subtype Analysis of a Predictive Model for Atrial Fibrillation in Patients With Hypertrophic Cardiomyopathy
Source: Rev Cardiovasc Med. 2025 Nov 27;26(11):45267. doi: 10.31083/RCM45267 (PMC12680985; doi:10.31083/RCM45267)
Supplement: Supplementary file 1 [file 2153-8174-26-11-45267-s1.docx]

| **Sequence Type** | **B-TFE cine sequence** | **T1 mapping**  **(modified MOLLI)** |
| --- | --- | --- |
| **Imaging Views/Planes** | Two-chamber, three-chamber, four-chamber long-axis views and contiguous short-axis slices of LV | Basal, midventricular, and apical short-axis planes |
| **TR (ms)** | 2.9 | 3.0–5.0 |
| **TE (ms)** | 1.47 | 1.0–1.5 |
| **FA (°)** | 45 | 30 |
| **Slice Thickness (mm)** | 8 | 8 |
| **Contrast Agent Dose** | None | 0.2 mmol/kg gadolinium-based |
| **Post-Injection Time** | - | 10–15 minutes |
| **Additional Parameters** | - | Breath-hold acquisition, anatomical alignment with cine sequences |

|  |
| --- |

**Supplementary Table 1: The parameters for CMR images**

**Supplementary Table 2: Missing Data from the Original Data**

| **Variable** | **Missing Count** | **Missing Ratio** |
| --- | --- | --- |
| Atrial fibrillation | 0 | 0.000 |
| CHD | 0 | 0.000 |
| Obstruction | 0 | 0.000 |
| Gender | 0 | 0.000 |
| Age | 0 | 0.000 |
| Heart rate | 0 | 0.000 |
| Hypertension | 0 | 0.000 |
| Diabetes | 0 | 0.000 |
| Alcohol | 0 | 0.000 |
| Smoking | 0 | 0.000 |
| BMI | 9 | 0.022 |
| NLR | 5 | 0.012 |
| Monocyte | 5 | 0.012 |
| Eosinophil | 5 | 0.012 |
| Basophil | 5 | 0.012 |
| Red blood cell | 4 | 0.010 |
| Hemoglobin | 1 | 0.002 |
| Platelet | 1 | 0.002 |
| **NT-proBNP** | **151** | **0.373** |
| **Troponin** | **55** | **0.136** |
| Alanine Aminotransferase | 3 | 0.007 |
| Aspartate Aminotransferase | 3 | 0.007 |
| Alkaline Phosphatase | 7 | 0.017 |
| Glutamyl Transpeptidase | 6 | 0.015 |
| Lactate Dehydrogenase | 7 | 0.017 |
| Direct Bilirubin | 8 | 0.020 |
| Cholinesterase | 8 | 0.020 |
| Albumin | 4 | 0.010 |
| Globulin | 7 | 0.017 |
| Leucine Aminopeptidase | 7 | 0.017 |
| Adenosine Deaminase | 7 | 0.017 |
| Urea | 6 | 0.015 |
| Creatinine | 5 | 0.012 |
| Triglyceride | 5 | 0.012 |
| HDL_C | 5 | 0.012 |
| LDL_C | 4 | 0.010 |
| C-reactive protein | 6 | 0.015 |
| eGFR | 5 | 0.012 |
| Thyroid Stimulating Hormone | 24 | 0.059 |
| Free Triiodothyronine | 25 | 0.062 |
| Free Thyroxine | 24 | 0.059 |
| **Thyroglobulin Antibody** | **244** | **0.602** |
| **Thyroid Peroxidase Antibody** | **293** | **0.723** |
| RAD Anteroposterior | 0 | 0.000 |
| LAD Anteroposterior | 1 | 0.002 |
| LVWT | 16 | 0.040 |
| IVST | 19 | 0.047 |
| LVEDV | 2 | 0.005 |
| LVESV | 0 | 0.000 |
| LVSV | 0 | 0.000 |
| LVEF | 1 | 0.002 |
| LVCO | 0 | 0.000 |
| LVCI | 9 | 0.022 |
| RVEDV | 0 | 0.000 |
| RVESV | 0 | 0.000 |
| RVSV | 0 | 0.000 |
| RVEF | 1 | 0.002 |
| RVCO | 0 | 0.000 |
| RVCI | 9 | 0.022 |
| MAPSE inferior | 3 | 0.007 |
| MAPSE Anterior | 3 | 0.007 |
| MAPSE Lateral | 1 | 0.002 |
| MAPSE Septal | 1 | 0.002 |
| TAPSE | 2 | 0.005 |
| LVGCS | 0 | 0.000 |
| LVGLS | 0 | 0.000 |
| LVGRS | 0 | 0.000 |
| RVGCS | 0 | 0.000 |
| RVGLS | 1 | 0.002 |
| RVGRS | 0 | 0.000 |
| MinLAV | 0 | 0.000 |
| MinLAA | 0 | 0.000 |
| MLAV | 0 | 0.000 |
| MaxLAA | 0 | 0.000 |
| LAEF | 1 | 0.002 |
| MinRAV | 1 | 0.002 |
| MinRAA | 1 | 0.002 |
| MRAV | 2 | 0.005 |
| MaxRAA | 2 | 0.005 |
| RAEF | 3 | 0.007 |
| **Pre-contrast T1** | **118** | **0.291** |
| **Post-contrast T1** | **147** | **0.363** |
| **Extracellular volume** | **154** | **0.380** |

CHD: Coronary Atherosclerotic Heart Disease; NRL: neutrophil-to-lymphocyteratio; HDL-C: High-Density Lipoprotein Cholesterol; LDL-C: Low-Density Lipoprotein Cholesterol; eGFR: estimated Glomerular Filtration Rate; LV: Left Ventricle; RV: Right Ventricle; LA: Left Atrium; RA: Right Atrium; RAD: Right Atrial Diameter; LAD: Left Atrial Diameter; LVWT: Left Ventricular Wall Thickness; IVST: Interventricular Septum Thickness; EDV: End-Diastolic Volume; ESV: End-Systolic Volume; SV: Stroke Volume; EF: Ejection Fractions; CO: Cardiac Output; CI: Cardiac Index; MAPSE: Mitral Annular Plane Systolic Excursion; TAPSE: Tricuspid Annular Plane Systolic Excursion; GCS: Global Circumferential Strain; GLS: Global Longitudinal Strain; GRS: Global Radial Strain; LAV: LA Volume; LAA: LA Area; RAV: RA Volume; RAA: RA Area.

**Supplementary Table 3: Highly Correlated Variables That Were Excluded and Their Correlation Coefficients**

| **Variable_1** | **Variable_2** | **Correlation_Coefficient** |
| --- | --- | --- |
| LVGCS | LVGRS | -0.9711634 |
| MinLAV | MinLAA | 0.8983296 |
| MinLAV | MaxLAV | 0.9245475 |
| MinLAA | MinLAV | 0.8983296 |
| MinLAA | MaxLAA | 0.9286594 |
| RVSV | RVCI | 0.8689950 |
| MinRAV | MaxRAV | 0.8812750 |
| RVGRS | RVGCS | -0.8481558 |
| LVSV | LVCO | 0.8392373 |
| LVCO | LVSV | 0.8392373 |
| LVCO | LVCI | 0.9190335 |

LV: Left Ventricle; RV: Right Ventricle; LA: Left Atrium; RA: Right Atrium; SV: Stroke Volume; EF: Ejection Fractions; CO: Cardiac Output; CI: Cardiac Index; GCS: Global Circumferential Strain; LAV: LA Volume; LAA: LA Area; RAV: RA Volume; RAA: RA Area.

**Supplementary Table 4：Baseline Characteristics and CMR Parameter Characteristics of the raining and Validation Sets**

| **Variable** | **Training Set** | **Validation Set** | **P_value** |
| --- | --- | --- | --- |
| AF, n (%) |  |  |  |
| Yes | 61 (21.4%) | 25 (20.8%) | 1.000 |
| CHD, n(%) |  |  |  |
| Yes | 44 (15.4%) | 29 (24.2%) | 0.052 |
| Obstruction, n(%) |  |  |  |
| Yes | 113 (39.6%) | 41 (34.2%) | 0.355 |
| Gender, n(%) |  |  | 0.415 |
| Female | 104 (36.5%) | 38 (31.7%) |  |
| Age | 57 (48-66) | 59 (48-68) | 0.494 |
| Heart rate (bpm) | 69 (63-76) | 68.5 (59-77.25) | 0.401 |
| Hypertension, n(%) |  |  |  |
| Yes | 151 (53%) | 67 (55.8%) | 0.677 |
| Diabetes, n(%) |  |  |  |
| Yes | 34 (11.9%) | 14 (11.7%) | 1.000 |
| Alcohol, n(%) |  |  |  |
| Yes | 48 (16.8%) | 27 (22.5%) | 0.231 |
| Smoking, n(%) |  |  |  |
| Yes | 86 (30.2%) | 43 (35.8%) | 0.318 |
| BMI | 25.86 ± 3.60 | 25.56 ± 3.70 | 0.451 |
| White blood cell (10^9/L) | 6.1 (5.1-7.3) | 6.1 (5.075-7.4) | 0.943 |
| NLR | 2.048 (1.6-2.903) | 1.975 (1.5-2.686) | 0.343 |
| Monocyte (10^9/L) | 0.4 (0.3-0.5) | 0.4 (0.3-0.5) | 0.278 |
| Eosinophil (10^9/L) | 0.13 (0.08-0.2) | 0.12 (0.07-0.213) | 0.310 |
| Basophil (10^9/L) | 0.03 (0.02-0.04) | 0.03 (0.02-0.04) | 0.945 |
| Red blood cell (10^12/L) | 4.57 (4.23-4.92) | 4.65 (4.21-5.04) | 0.478 |
| Hemoglobin (g/L) | 140 (128-151) | 141 (128.8-154.2) | 0.413 |
| Platelet (10^9/L) | 191 (151-231) | 181.5 (148.5-222) | 0.264 |
| Alanine Aminotransferase (U/L) | 19.9 (14.5-30.9) | 20.2 (15.05-26.47) | 0.976 |
| Aspartate Aminotransferase (U/L) | 21.2 (17.9-25.9) | 20.8 (17.58-25.69) | 0.921 |
| Alkaline Phosphatase (U/L) | 69 (56-80.3) | 71.05 (56.72-81.2) | 0.485 |
| Glutamyl Transpeptidase (U/L) | 27.6 (18.7-41.8) | 28 (19.38-48.1) | 0.662 |
| Lactate Dehydrogenase (U/L) | 201 (179-235) | 197 (168.8-231.2) | 0.270 |
| Direct Bilirubin (umol/L) | 2.4 (1.8-3.5) | 2.45 (1.7-3.2) | 0.391 |
| Cholinesterase (KU/L) | 8 (6.9-9.1) | 7.7 (6.6-9.1) | 0.192 |
| Albumin (g/L) | 40.8 (38.8-42.4) | 40.75 (38.4-42.28) | 0.486 |
| Globulin (g/L) | 25 (22-27.6) | 24.65 (21.67-26.95) | 0.573 |
| Leucine Aminopeptidase (U/L) | 43.25 (29.4-52) | 42.6 (30.3-50.85) | 0.901 |
| Adenosine Deaminase (U/L) | 9.7 (7.8-11.8) | 10 (7.7-13.1) | 0.426 |
| Urea (mmol/L) | 6 (4.9-7.1) | 6.15 (5-7.025) | 0.919 |
| Creatinine (umol/L) | 69 (58-80) | 71 (59-80.25) | 0.408 |
| Triglyceride (mmol/L) | 1.27 (0.91-1.8) | 1.225 (0.9-1.67) | 0.439 |
| HDL-C (mmol/L) | 1.06 (0.9-1.23) | 1.075 (0.9075-1.272) | 0.721 |
| LDL-C (mmol/L) | 2.44 (2.05-3.02) | 2.415 (1.875-2.95) | 0.400 |
| C-reactive protein (mg/L) | 3.5 (2.2-5.5) | 3.5 (2.4-5.175) | 0.978 |
| eGFR (mL/min) | 101.2 (85.6-118.9) | 100.9 (82.15-116.4) | 0.668 |
| TSH (mLU/L) | 1.99 (1.23-2.82) | 2.005 (1.472-2.61) | 0.429 |
| Free Triiodothyronine (pmoL/L) | 4.65 (4.19-5.07) | 4.715 (4.28-5.22) | 0.306 |
| Free Thyroxine (pmoL/L) | 16.51 (14.6-18.1) | 16.5 (14.97-17.72) | 0.869 |
| **CMR parameters** |  |  |  |
| RAD Anteroposterior (cm) | 3.3 (2.9-3.9) | 3.3 (3-3.8) | 0.973 |
| LAD Anteroposterior (cm) | 3.3 (2.8-4) | 3.2 (2.7-3.8) | 0.201 |
| LVWT (cm) | 1 (0.8-1.2) | 0.9 (0.8-1.1) | 0.084 |
| IVST (cm) | 1.9 (1.6-2.3) | 1.85 (1.6-2.3) | 0.839 |
| LVEDV (mL) | 130.6 (112.1-153.7) | 130.1 (112.5-156.1) | 0.765 |
| LVESV (mL) | 52.98 (40.33-68.08) | 56.41 (43.21-67.22) | 0.335 |
| LVEF | 58.69 (50.36-64.46) | 58.2 (52.13-64.59) | 0.998 |
| LVCI (L/min/m2) | 2.74 (2.27-3.27) | 2.73 (2.215-3.208) | 0.775 |
| RVEDV (mL) | 112.3 (95.07-137.4) | 111.7 (90.94-134.9) | 0.444 |
| RVESV (mL) | 57.86 (44.1-74.59) | 55.47 (44.48-66.12) | 0.360 |
| RVEF | 50.08 (38.75-57.7) | 51.11 (35.34-59.81) | 0.707 |
| RVCO (L/min) | 3.76 (2.8-4.82) | 3.495 (2.475-4.697) | 0.388 |
| RVCI (L/min/m2) | 2.068 (1.53-2.61) | 1.99 (1.407-2.672) | 0.567 |
| MAPSE inferior (mm) | 9.602 ± 3.52 | 10.54 ± 4.26 | **0.035** |
| MAPSE Anterior (mm) | 8.99 (6.91-11.18) | 10.23 (7.295-12.31) | **0.016** |
| MAPSE Lateral (mm) | 12.5 (10.02-14.72) | 13.21 (10.07-15.36) | 0.296 |
| MAPSE Septal (mm) | 8.84 (6.21-10.62) | 9.61 (7.535-11.53) | **0.034** |
| TAPSE (mm) | 17.58 (13.72-21.7) | 17.98 (13.83-21.47) | 0.738 |
| LVGLS | -11.8 (-14.5--9.1) | -12.35 (-14.72--8.8) | 0.784 |
| LVGRS | 27.98 ± 9.385 | 27.37 ± 9.482 | 0.551 |
| RVGCS | -13.5 (-16.1--10.49) | -13.31 (-16.34--10.47) | 0.819 |
| RVGLS | -23.15 (-26.34--18.67) | -23.96 (-26.77--18.41) | 0.808 |
| MaxLAV (mL) | 84.33 (62.34-109.5) | 83.21 (62.79-109.4) | 0.819 |
| MaxLAA (cm²) | 24.75 (20.61-29.37) | 23.77 (20.28-29) | 0.711 |
| LAEF | 49.75 (39.21-57.01) | 49.69 (41.22-58.92) | 0.600 |
| MinRAA (cm²) | 11.47 (9.117-14.27) | 11.74 (8.911-14.68) | 0.649 |
| MaxRAV (mL) | 57.47 (45.64-73.76) | 59.03 (44.35-74.13) | 0.906 |
| MaxRAA (cm²) | 19.02 (16.4-22.5) | 19.02 (16.79-23.09) | 0.790 |
| RAEF | 49.13 ± 14.17 | 47.71 ± 15.26 | 0.384 |

CHD: Coronary Atherosclerotic Heart Disease; NRL: neutrophil-to-lymphocyteratio; HDL-C: High-Density Lipoprotein Cholesterol; LDL-C: Low-Density Lipoprotein Cholesterol; eGFR: estimated Glomerular Filtration Rate; TSH: Thyroid Stimulating Hormone; LV: Left Ventricle; RV: Right Ventricle; LA: Left Atrium; RA: Right Atrium; RAD: Right Atrial Diameter; LAD: Left Atrial Diameter; LVWT: Left Ventricular Wall Thickness; IVST: Interventricular Septum Thickness; EDV: End-Diastolic Volume; ESV: End-Systolic Volume; EF: Ejection Fractions; CO: Cardiac Output; CI: Cardiac Index; MAPSE: Mitral Annular Plane Systolic Excursion; TAPSE: Tricuspid Annular Plane Systolic Excursion; GCS: Global Circumferential Strain; GLS: Global Longitudinal Strain; GRS: Global Radial Strain; LAV: LA Volume; LAA: LA Area; RAV: RA Volume; RAA: RA Area.

**Supplementary Table 5: Baseline Characteristics and CMR Parameter Characteristics of HOCM and HNCM**

| **Variable** | **HOCM (n=154）** | **HNCM (n=251)** | **P_value** |
| --- | --- | --- | --- |
| AF, n (%) |  |  | 0.423 |
| No | 125 (81.2%) | 194 (77.3%) |  |
| Yes | 29 (18.8%) | 57 (22.7%) |  |
| CHD, n (%) |  |  | 0.548 |
| No | 129 (83.8%) | 203 (80.9%) |  |
| Yes | 25 (16.2%) | 48 (19.1%) |  |
| Gender, n (%) |  |  | **0.042** |
| Female | 64 (41.6%) | 78 (31.1%) |  |
| Male | 90 (58.4%) | 173 (68.9%) |  |
| Age | 57 (50-66) | 57 (46-67) | 0.961 |
| Heart rate (bpm) | 68 (62-75) | 69 (62-78) | 0.468 |
| Hypertension, n (%) |  |  | 1.000 |
| No | 71 (46.1%) | 116 (46.2%) |  |
| Yes | 83 (53.9%) | 135 (53.8%) |  |
| Diabetes, n (%) |  |  | 0.937 |
| No | 135 (87.7%) | 222 (88.4%) |  |
| Yes | 19 (12.3%) | 29 (11.6%) |  |
| Alcohol, n (%) |  |  | 0.186 |
| No | 131 (85.1%) | 199 (79.3%) |  |
| Yes | 23 (14.9%) | 52 (20.7%) |  |
| Smoking, n (%) |  |  | 0.733 |
| No | 107 (69.5%) | 169 (67.3%) |  |
| Yes | 47 (30.5%) | 82 (32.7%) |  |
| BMI | 25.78 (23.66-28.07) | 25.71 (23.05-28.02) | 0.254 |
| White blood cell (10^9/L) | 6 (5.1-7.4) | 6.1 (5.15-7.4) | 0.949 |
| NLR | 2.022 (1.588-2.911) | 2 (1.551-2.795) | 0.837 |
| Monocyte (10^9/L) | 0.4 (0.3-0.5) | 0.4 (0.3-0.5) | 0.650 |
| Eosinophil (10^9/L) | 0.13 (0.09-0.2) | 0.13 (0.07-0.21) | 0.303 |
| Basophil (10^9/L) | 0.03 (0.02-0.04) | 0.03 (0.02-0.04) | 0.891 |
| Red blood cell (10^12/L) | 4.51 (4.14-4.878) | 4.62 (4.32-5.01) | 0.062 |
| Hemoglobin (g/L) | 139 (127.2-150) | 141 (129-154) | 0.152 |
| Platelet (10^9/L) | 188 (153.2-231.2) | 188 (149-226) | 0.552 |
| Alanine Aminotransferase (U/L) | 19 (14.35-26.98) | 20.7 (15-30.8) | 0.183 |
| Aspartate Aminotransferase (U/L) | 20.05 (17.1-25.25) | 21.6 (18.3-26.75) | 0.050 |
| Alkaline Phosphatase (U/L) | 70 (57.75-81.2) | 67.7 (56.1-80.25) | 0.572 |
| Glutamyl Transpeptidase (U/L) | 26.95 (18.15-43.42) | 28.5 (19.35-44.5) | 0.572 |
| Lactate Dehydrogenase (U/L) | 207 (176.5-245) | 197 (175.5-230) | 0.137 |
| Direct Bilirubin (umol/L) | 2.3 (1.8-3.1) | 2.5 (1.8-3.6) | 0.053 |
| Cholinesterase (KU/L) | 7.85 (6.9-8.9) | 7.9 (6.7-9.2) | 0.986 |
| Albumin (g/L) | 40.5 (38.62-41.98) | 40.9 (38.75-42.6) | 0.075 |
| Globulin (g/L) | 25 (22.13-27.55) | 24.7 (21.85-27.3) | 0.737 |
| Leucine Aminopeptidase (U/L) | 43.13 (32.92-50.88) | 43.25 (29.15-52) | 0.896 |
| Adenosine Deaminase (U/L) | 9.7 (7.8-11.78) | 9.7 (7.75-12.4) | 0.702 |
| Urea (mmol/L) | 6.1 (4.9-7.2) | 6 (4.9-7) | 0.679 |
| Creatinine (umol/L) | 66 (56-77.75) | 72 (61-81) | **0.004** |
| Triglyceride (mmol/L) | 1.275 (0.9025-1.825) | 1.25 (0.91-1.745) | 0.625 |
| HDL-C (mmol/L) | 1.08 (0.91-1.248) | 1.06 (0.9-1.25) | 0.922 |
| LDL-C (mmol/L) | 2.628 ± 0.7263 | 2.432 ± 0.8349 | **0.013** |
| C-reactive protein (mg/L) | 3.7 (2.425-5.6) | 3.5 (2.2-5.35) | 0.407 |
| eGFR (mL/min) | 101.9 (88.4-119.5) | 100.5 (80.35-117.6) | 0.161 |
| TSH (mLU/L) | 2.035 (1.263-2.808) | 1.95 (1.37-2.74) | 0.848 |
| Free Triiodothyronine (pmoL/L) | 4.73 (4.245-5.132) | 4.63 (4.195-5.11) | 0.718 |
| Free Thyroxine (pmoL/L) | 16.51 (15.1-18.1) | 16.5 (14.65-18.09) | 0.421 |
| **CMR parameters** |  |  |  |
| RAD Anteroposterior (cm) | 3.3 (3-3.8) | 3.3 (3-4) | 0.261 |
| LAD Anteroposterior (cm) | 3.4 (3-3.975) | 3.2 (2.7-3.8) | 0.088 |
| LVWT (cm) | 0.9 (0.8-1.2) | 1 (0.8-1.2) | 0.679 |
| IVST (cm) | 2 (1.7-2.3) | 1.8 (1.5-2.2) | **0.006** |
| LVEDV (mL) | 131.3 (111.2-153.3) | 128.9 (113.3-156.3) | 0.692 |
| LVESV (mL) | 53.12 (40.42-63.83) | 54.69 (41.45-70.13) | 0.110 |
| LVEF | 60.11 (53.95-65.12) | 57.22 (48.33-64.07) | 0.004 |
| LVCI (L/min/m2) | 2.77 (2.48-3.415) | 2.68 (2.17-3.205) | **0.013** |
| RVEDV (mL) | 107.8 (94.99-129.1) | 116.8 (93.6-138.8) | 0.117 |
| RVESV (mL) | 54.55 (43.58-65.86) | 58.58 (44.61-74.65) | 0.060 |
| RVEF | 50.8 (40.14-58.76) | 49.83 (35.55-57.69) | 0.225 |
| RVCO (L/min) | 3.58 (2.792-4.605) | 3.79 (2.575-4.925) | 0.999 |
| RVCI (L/min/m2) | 2.055 (1.562-2.605) | 2.05 (1.425-2.655) | 0.691 |
| MAPSE inferior (mm) | 10.29 (8.025-13.23) | 9.33 (6.67-11.98) | **0.005** |
| MAPSE Anterior (mm) | 9.645 (7.212-11.54) | 9.09 (6.9-11.57) | 0.293 |
| MAPSE Lateral (mm) | 13.5 (11-15.56) | 12.1 (9.705-14.55) | **0.004** |
| MAPSE Septal (mm) | 9.38 (7.487-11.3) | 8.71 (6.41-11.01) | 0.082 |
| TAPSE (mm) | 18.51 ± 6.256 | 17.05 ± 5.987 | **0.021** |
| LVGLS | -11.96 (-14.7--9.773) | -11.8 (-14.5--8.48) | 0.113 |
| LVGRS | 29.52 ± 8.517 | 26.74 ± 9.78 | **0.003** |
| RVGCS | -13.47 (-15.71--10.65) | -13.44 (-16.55--10.3) | 0.746 |
| RVGLS | -23.64 (-26.5--19.13) | -22.99 (-26.67--18.32) | 0.464 |
| MaxLAV (mL) | 90.82 (66.93-114.3) | 77.4 (60.59-106.2) | **0.006** |
| MaxLAA (cm²) | 25.42 (21.51-29.78) | 23.33 (20.12-28.83) | **0.016** |
| LAEF | 49.09 (41.77-55.48) | 50.14 (37.14-58.92) | 0.570 |
| MinRAA (cm²) | 11.28 (8.981-14.14) | 11.74 (9.193-14.64) | 0.345 |
| MaxRAV (mL) | 57.45 (47.25-70.83) | 58.95 (43.84-76.72) | 0.853 |
| MaxRAA (cm²) | 19.05 (16.99-22.23) | 19.02 (16.2-22.98) | 0.912 |
| RAEF | 49.81 (39.1-58.58) | 47.08 (39.69-55.72) | 0.194 |

CHD: Coronary Atherosclerotic Heart Disease; NRL: neutrophil-to-lymphocyteratio; HDL-C: High-Density Lipoprotein Cholesterol; LDL-C: Low-Density Lipoprotein Cholesterol; eGFR: estimated Glomerular Filtration Rate; TSH: Thyroid Stimulating Hormone; LV: Left Ventricle; RV: Right Ventricle; LA: Left Atrium; RA: Right Atrium; RAD: Right Atrial Diameter; LAD: Left Atrial Diameter; LVWT: Left Ventricular Wall Thickness; IVST: Interventricular Septum Thickness; EDV: End-Diastolic Volume; ESV: End-Systolic Volume; EF: Ejection Fractions; CO: Cardiac Output; CI: Cardiac Index; MAPSE: Mitral Annular Plane Systolic Excursion; TAPSE: Tricuspid Annular Plane Systolic Excursion; GCS: Global Circumferential Strain; GLS: Global Longitudinal Strain; GRS: Global Radial Strain; LAV: LA Volume; LAA: LA Area; RAV: RA Volume; RAA: RA Area.


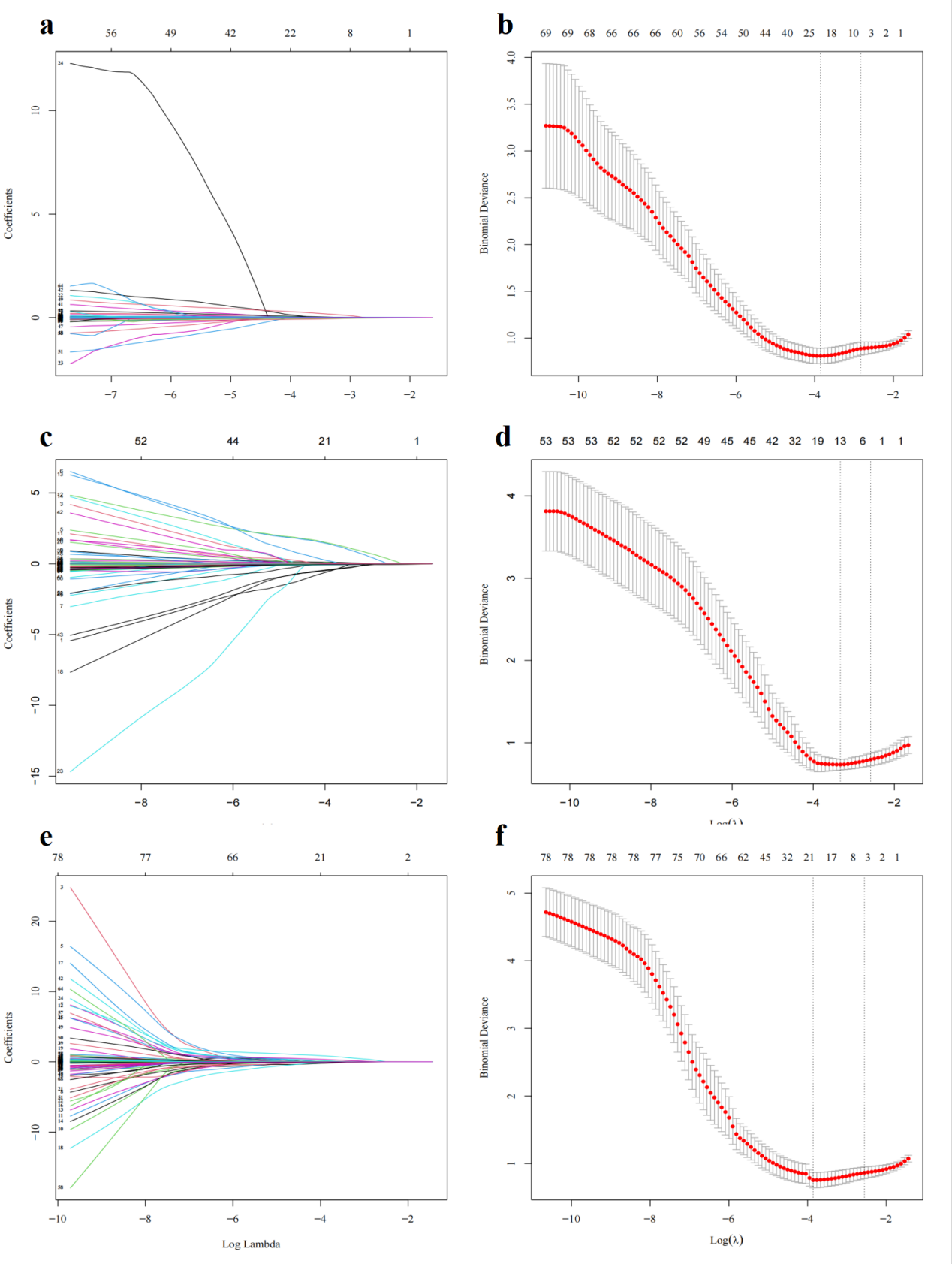


**Supplementary Fig. 1：Lasso regression.** Training Set: (a)Relationship between log(λ) and lasso regression coefficients; (b) Lasso regression with 10-fold cross-validation；HOCM: (c) Relationship between log(λ) and lasso regression coefficients;(d) Lasso regression with 10-fold cross-validation; HNCM: (e) Relationship between log(λ) and lasso regression coefficients; (f) Lasso regression with 10-fold cross-validation;
